# Data From the NeFeLaE Project: “Cross-Cultural Evaluations of Grammatically Gendered Occupational Terms: Gender Representations From French and Brazilian Adults”

**DOI:** 10.5334/jopd.146

**Published:** 2026-11-12

**Authors:** Morgane Burnel, Catherine Esnard, Rebeca Grangeiro, Lauriane Véron-Delor

**Affiliations:** 1 Université de Poitiers, Centre de recherches sur la Cognition et l’Apprentissage (CeRCA), Université de Poitiers, France; 2 Laboratoire Parisien de Psychologie Sociale (LAPPS), Université Paris Nanterre, France

**Keywords:** Gender-fair language, Gendered occupations, French, Portuguese, Representations

## Abstract

This dataset contains gender representations ratings for 56 occupations in both French and Brazilian Portuguese. Data were collected online from adult participants in France (N = 116) and Brazil (N = 110). Participants rated how feminine or masculine each occupation is perceived in 2024, using a Likert scale. The study focuses on occupations with distinct masculine and feminine grammatical forms in both French and Brazilian Portuguese. Data, codebook, survey instruments, and analysis scripts are stored on OSF. The dataset is reusable in sociolinguistics, psycholinguistics, gender studies, and cross-cultural research.

## (1) Background

This study aimed to compile a list of grammatically gendered occupations associated with gender stereotypes in both French and Brazilian Portuguese. To achieve this, participants were asked to evaluate how masculine or feminine different occupations are generally perceived. The aim was to develop a set of experimental materials for a subsequent study examining the impact of gender-fair language on the mental representation of women in groups associated with gendered occupations.

In grammatical-gender languages such as French and Portuguese, the masculine plural is the default form used to refer to mixed or unspecified groups, even when a single male is present among a large number of women. Unlike the feminine, which always refers to an exclusively female group, the masculine form may carry either a specific meaning (i.e., referring to a group of men only) or a generic one (i.e., referring to a mixed-gender group or a group whose gender composition is unknown). The ‘generic masculine’ introduces semantic ambiguity regarding people’s gender within a group. For instance, the sentence *“Les médecins sont arrivés”* (i.e., “The doctors have arrived”) can refer either to an all-male group or to a mixed-gender group, with no grammatical indication to disambiguate the reference.

This ambiguity between the specific and the generic use of masculine constitutes a real challenge for language processing. Specifically, when masculine forms are used, listeners and readers must rely on context or world knowledge to determine whether the group referred to is all-male or gender-mixed. However, research suggests that this ambiguity is rarely resolved in a neutral manner. In fact, it is interpreted as male-specific by default, thereby contributing to the reduced presence of women in mental representations (Gygax et al., 2019). Furthermore, gender stereotypes are socially shared expectations linking certain social or professional roles more strongly to one gender than the other (Misersky et al., 2014). Thus, when individuals encounter occupational or role names, these associations can be activated automatically, as shown in both social psychology (Banaji & Hardin, 1996) and psycholinguistics (Oakhill et al., 2005). In grammatical-gender languages, generic masculine forms reinforce this effect. When roles are presented in the masculine plural, they evoke male representations, even when the intended reference is mixed-gender. For example, Gygax and Gabriel (2008) found that French-speaking participants were more likely to associate feminine role nouns (such as nurse) with masculine terms (like brother) than with feminine ones (like sister), suggesting a male-specific interpretation of the supposedly generic form, regardless of the gender stereotype typically associated with the occupation. Similarly, judgments about the proportion of women in groups described by gender-neutral or weakly stereotyped masculine nouns frequently fall below parity, even in the absence of contextual cues (Tibblin et al., 2023).

Repeated exposure to the generic masculine also affects more subjective dimensions of social representation. For example, in a study involving adolescents, using masculine-only forms to designate professions reduced girls’ expressed confidence in their ability to succeed in those fields (Chatard et al., 2005). These effects indicate that gendered representations are not limited to implicit cognitive associations, but also influence how individuals relate to roles and project themselves into specific social or professional domains. In addition, when role names are evaluated in terms of stereotypicality, the exclusive use of masculine forms tends to increase the salience of the male gender. For example, Gabriel et al. (2008) report that using masculine forms to label professions leads to a perception bias, making these roles seem more male-dominated than they actually are.

In response to this systematic activation of male-specific representations, several studies have examined whether gender-fair language strategies can reduce the bias introduced by ‘generic’ masculine. Two main approaches are typically distinguished. First, neutralization involves using gender-unmarked forms (e.g., *la direction_[neutr.]_* | directors). Second, refeminization reintroduces feminine markers through double forms (e.g., *les directeurs_[masc.]_ et les directrices_[fem.]_* | directors). Although findings are sometimes mixed, a number of studies suggest that double forms promote more gender-balanced mental representations than neutral ones (Brauer & Landry, 2008; Gabriel et al., 2018; Spinelli et al., 2023; Vervecken et al., 2015). Moreover, this effect appears particularly pronounced when these forms are applied to occupations associated with strong gender stereotypes (Irmen & Roßberg, 2004; Tibblin et al., 2023).

The present study follows the refeminization approach, focusing on grammatically gendered occupational role nouns rather than gender-unmarked or generic noun forms (see Gygax et al., 2019). Accordingly, we selected occupational terms whose grammatical gender is explicitly marked, with distinct masculine and feminine forms. The focus on occupations where the feminine form of the noun is phonologically and orthographically distinct from the masculine form (e.g., *avocat_[masc.]_* vs. *avocate_[fem.]_* | lawyer) allows for the explicit perception of grammatical gender, which is essential for investigating the potential cognitive effects of refeminization strategies.

Based on Gabriel et al. (2008), Misersky et al. (2014) provided an exceptionally rich set of role nouns for research on gender stereotypes. Based on data collected from 1,663 adults, this database provides gender stereotypicality norms for more than 400 social and occupational role nouns across seven languages, including French but not Portuguese. However, several limitations should be noted: the data for French language were collected in Switzerland, where gender stereotypes may differ from those observed in France; the norms were established more than a decade ago and may therefore not fully reflect current stereotypes; and the sample size was relatively modest (approximately N = 32 per role noun).

Building on Misersky et al. (2014), Spinelli et al. (2023) developed a complementary database for France following the neutralization approach (i.e., focusing on gender-unmarked role nouns selected to be as gender-neutral as possible). In contrast, the present study follows the refeminization approach and therefore requires different selection criteria. Most of the occupations retained in the present study were drawn from the database developed by Misersky et al. (2014), which provided a broad set of role nouns suitable for studying gender stereotypes. However, given the specific objectives of the present study, we selected occupational terms that (1) were associated with gender stereotypes, and (2) had distinct masculine and feminine forms (3) in both French and Brazilian Portuguese.

Following Misersky et al. (2014), participants were instructed to estimate how occupations are perceived rather than expressing personal preferences or beliefs about how occupations should ideally be distributed across genders. However, whereas previous studies assessed perceived gender ratios as an indirect indicator of occupational gender stereotypicality, the present study directly assessed perceived occupational gender stereotypes, that is, the extent to which occupations are perceived as more masculine or feminine. Asking participants to estimate how occupations are generally perceived may also reduce the influence of individual opinions, values, or experiences. This approach is particularly relevant when studying gender stereotypes, as participants’ personal attitudes may differ from the broader social representations they aim to estimate, especially in samples that may not perfectly reflect the diversity of the general population.

A continuous rating scale (from 0 to 100) was used because it allows for a fine-grained assessment of gradual variations in occupational gender stereotypes. Rather than imposing categorical distinctions between masculine, neutral, and feminine occupations, this continuous approach captures the extent to which occupations are perceived as positioned along a masculinity-femininity continuum. Special attention was also given to the orientation of the response scale, which was counterbalanced across participants to control for potential anchoring effects (Gabriel et al., 2008; Misersky et al., 2014).

To summarize, building on Gabriel et al. (2008) and Misersky et al. (2014), the present study focuses on occupational terms that (1) were associated with gender stereotypes, and (2) had distinct masculine and feminine forms (3) in both French and Brazilian Portuguese. The present study built on the methodological approach of previous paradigms while adapting the scale interpretation from perceived gender ratios to perceived occupational genderedness along a masculinity-femininity continuum.

## (2) Methods

### 2.1 Study design

The goal of the study was to identify occupations associated with gender stereotypes in France and Brazil. Based on linguistic criteria, namely the presence of grammatically gendered forms in both French and Brazilian Portuguese, 56 occupations were selected, mostly from Misersky et al. (2014).

The study employed a mixed design. Language (French vs. Brazilian Portuguese) was treated as a between-participants factor, whereas occupational ratings were collected using a within-participants design within each language group. The independent variable was occupation (e.g., dancer | *dançarino* | *danseur*), and the dependent variable was the perceived femininity rating assigned to each occupation by participants.

Data were collected through an online questionnaire administered using Qualtrics software. Participants were instructed to estimate how they believed each occupation, presented in its grammatical masculine form, was socially perceived in 2024. They answered the following question: “In your opinion, in 2024, is the job of [occupation_[masc.]_] perceived as rather *masculine* or rather *feminine*?”.

Responses were collected using a horizontal slider ranging from 0 to 100, with a step size of 1. The midpoint of the scale was labelled as “neutral”, while the two endpoints were labelled “feminine” and “masculine”. To control for potential anchoring effects, the position of the feminine and masculine labels on the left and right sides of the scale was counterbalanced across participants, as was the order of the response options in the question (i.e., “rather feminine or rather masculine” vs. “rather masculine or rather feminine”). To provide their response, participants were required to move the cursor from its initial central position. While moving the cursor, the corresponding numerical value was displayed, ranging from 0 at the left end of the scale to 100 at the right end of the scale.

Before data analysis, scores obtained from scales oriented from feminine to masculine were reverse-coded to place all responses on a common metric. All data and results are therefore presented as perceived femininity ratings, with scores closer to 100 indicating more feminine-gendered representations, scores closer to 0 indicating more masculine-gendered representations, and scores around 50 indicating occupations perceived as less gender-stereotyped.

### 2.2 Time of data collection

The data was collected from June to September 2024.

### 2.3 Location of data collection

Data was collected online using Qualtrics. French-speaking participants were recruited in France through the CNRS (Cognitive Sciences Information Network, RISC) “Expésciences” volunteer list. Portuguese-speaking participants were recruited in Brazil from academia. The recruitment advertisements explicitly targeted native French speakers living in France and native Portuguese speakers living in Brazil.

### 2.4 Sampling, sample and data collection

A total of 236 Brazilian and French people initially participated in the study. However, 10 participants were excluded because concerns were raised regarding the reliability of their responses (see Section 2.6). The results presented below are therefore based on a final sample of 116 French participants (male = 50, female = 60, non-binary = 6) and 110 Brazilian participants (male = 51, female = 58, non-binary = 1). The mean age was 26.06 (σ = 8.9) for Brazilians, and 38.89 (σ = 18.5) for French. According to a Welch’s t-test, French participants were statistically significantly older than Brazilian participants (t(167.21) = –6.70, p < .001).

Participants did not receive compensation. There were no missing data, as only complete responses were recorded by the data collection software. No additional information was collected regarding participants’ country of origin, length of residence, language proficiency, or bilingual/multilingual status. Therefore, native status and language proficiency were not formally verified beyond the recruitment criteria stated in the advertisement.

### 2.5 Materials/Survey instruments

The 56 occupational role nouns tested were pre-selected using criteria of (1) being masculine- or feminine-stereotyped (based on previous research by Misersky et al., 2014), and (2) being grammatically gendered role nouns with distinct masculine and feminine forms in both French and Portuguese.

The exact materials used and Qualtrics scripts are available at DOI: osf.io/j86z4.

### 2.6 Quality Control

To identify participants who may not have followed the instructions, either by providing nearly identical ratings across all occupations or by producing unusually extreme response patterns, we conducted two complementary quality checks in each group of participants.

First, we calculated the within-participant standard deviation (SD) across the 56 occupations. Participants whose within-participant SD was more than 2.5 SD from the country-specific sample mean were considered to show insufficient response variability (e.g., consistently giving the same or very similar ratings across occupations) or excessively variable response patterns.

Next, for each occupation, we computed the mean rating and SD within the country-specific sample. We then identified deviant responses, defined as ratings located more than 2.5 SD from the occupation-specific mean. For each participant, we counted the number of such deviant responses and calculated the (country-specific) sample mean of this count. Participants whose number of deviant responses exceeded 2.5 SD above the sample mean were excluded from further analyses, as this pattern is consistent with either largely random responding or a systematic tendency to provide ratings that diverged markedly from the responses observed in the rest of the sample.

In total, 10 participants were excluded, five from the French sample and five from the Brazilian sample.

In the remaining samples of 116 French participants and 110 Brazilian participants, we first conducted two within-language one-tailed correlations comparing the mean perceived femininity ratings obtained under the two scale directions for the 56 occupations. The results were statistically significant in both French (r_Pearson_ = .99, p < .001) and Brazilian participants (r_Pearson_ = .98, p < .001), showing that the two scale orientations produced highly convergent evaluations in each sample.

Then, we conducted a between-language one-tailed correlation on the mean perceived femininity ratings for the 56 occupations tested. The result was statistically significant (r_Spearman_ = .88, p < .001), showing a general tendency for gender representations to be similar across France and Brazil. Detailed perceived femininity ratings (means and standard deviations) for each occupation in each country are presented in Table 1.

**Table 1:** **Wilcoxon signed-rank tests comparing perceived femininity ratings to the midpoint (50%) for each occupation in the French (N = 116) and Brazilian (N = 110) samples**. Statistically significant deviations from the midpoint indicated gender-stereotyped occupations: higher scores reflected stronger feminine associations, whereas lower scores reflected stronger masculine associations. Non-significant deviations indicated occupations with relatively weak gender-stereotypical associations.


GENDER STEREOTYPE FRANCE/BRAZIL	OCCUPATIONS	FRANCE				BRAZIL			
	
		MEAN (SD)	CORR.	V	p	MEAN (SD)	CORR.	V	p

Feminine/Feminine	caregiver	65.08 (14.51)	0.26	4267.0	<.001	74.28 (21.22)	0.38	4211.0	<.001

	childcare worker	80.83 (17.83)	0.87	6228.0	<.001	74.33 (21.41)	0.37	4118.0	<.001

	childminder	87.37 (12.25)	1.00	6786.0	<.001	86.13 (17.34)	0.68	5126.5	<.001

	dancer	64.87 (15.02)	0.18	3990.5	<.001	70.65 (20.97)	0.24	3728.5	<.001

	decorator	64.78 (15.62)	0.44	4885.0	<.001	70.89 (22.32)	0.17	3520.0	<.001

	dressmaker	61.91 (21.77)	0.31	4359.5	<.001	81.46 (18.32)	0.70	5194.0	<.001

	hairdresser	66.37 (15.97)	0.45	4922.0	<.001	58.84 (27)	–0.18	2327.0	<.001

	influencer	58.94 (15.83)	–0.23	2622.5	<.001	64.43 (19.89)	–0.27	2215.5	<.001

	makeup artist	79.03 (14.43)	1.00	6774.0	<.001	80.95 (21.03)	0.53	4654.0	<.001

	nurse	72.86 (13.71)	0.82	6190.0	<.001	69.91 (20.58)	0.17	3572.5	<.001

	pharmacist	52.86 (13.23)	–0.17	2758.5	.008	56.76 (21.03)	–0.38	1831.0	.002

	salesperson	56.05 (11.95)	–0.29	2421.0	<.001	54.2 (18.21)	–0.59	1232.0	.040

	teacher	60.95 (14.18)	0.14	3877.0	<.001	56.34 (17.87)	–0.50	1501.0	<.001

Masculine/Masculine	advisor	45 (13.6)	–0.82	600.5	<.001	41.28 (19.36)	–0.76	620.5	<.001

	aviator	23.22 (14.95)	–0.98	63.5	<.001	19.11 (18.82)	–0.98	39.0	<.001

	banker	32.09 (15.49)	–0.99	26.5	<.001	26.46 (20.38)	–0.96	77.5	<.001

	beekeeper	34.93 (14.5)	–0.98	51.0	<.001	25.09 (20.41)	–0.95	107.5	<.001

	butcher	17.31 (12.75)	–1.00	0.0	<.001	12.82 (16.53)	–0.99	13.0	<.001

	caretaker	29.83 (20.64)	–0.88	354.0	<.001	27.93 (25.52)	–0.75	504.5	<.001

	delivery person	16.78 (12.98)	–1.00	0.0	<.001	12.44 (14.76)	–1.00	1.0	<.001

	director	31.78 (13.59)	–0.99	20.0	<.001	30.68 (18.2)	–0.98	47.5	<.001

	doctor	45.42 (10.7)	–0.78	738.5	<.001	41.82 (17.51)	–0.82	489.5	<.001

	farmer	27.22 (14.27)	–1.00	11.5	<.001	23.78 (20.28)	–0.96	79.5	<.001

	firefighter	20.89 (14.49)	–1.00	4.0	<.001	17.5 (16.42)	–1.00	0.0	<.001

	footballer	16.77 (12.98)	–1.00	1.0	<.001	18.8 (19.4)	–0.97	49.5	<.001

	gardener	28.31 (14.57)	–0.98	52.0	<.001	40.76 (33.53)	–0.24	1763.0	.009

	governor	24.3 (16.4)	–0.99	37.0	<.001	25.79 (19)	–0.98	38.0	<.001

	horticulturist	40.15 (16.43)	–0.77	714.0	<.001	33.49 (21.92)	–0.87	303.5	<.001

	hotelier	44.47 (12.49)	–0.88	382.0	<.001	38.28 (21.46)	–0.79	519.0	<.001

	hunter	9.76 (9.96)	–1.00	0.0	<.001	11.99 (15.99)	–0.97	41.5	<.001

	inspector	33.45 (13.79)	–0.98	78.0	<.001	27.75 (21.2)	–0.90	215.5	<.001

	inventor	31.42 (14.88)	–0.99	44.5	<.001	34.45 (17.46)	–0.96	107.5	<.001

	jeweler	44.92 (17.67)	–0.64	1134.5	.004	34.32 (25.41)	–0.73	623.5	<.001

	lawyer	44.74 (13.4)	–0.78	721.0	<.001	40.58 (17.54)	–0.88	324.0	<.001

	lumberjack	8.72 (9.96)	–1.00	0.0	<.001	10.82 (13.98)	–1.00	3.0	<.001

	magistrate	39.39 (18.95)	–0.76	740.0	<.001	38.77 (21.5)	–0.76	601.5	<.001

	manager	37.52 (14.54)	–0.91	277.5	<.001	42.16 (19.41)	–0.81	503.0	<.001

	mayor	35.35 (13.87)	–0.99	28.5	<.001	27.32 (20.5)	–0.95	102.5	<.001

	musician	44.7 (12.33)	–0.87	436.5	<.001	40.99 (17.23)	–0.93	210.0	<.001

	programmer	23.37 (15.91)	–0.99	21.5	<.001	25.55 (21.19)	–0.92	171.5	<.001

	publisher	41.63 (15.01)	–0.83	554.0	<.001	37.33 (19.69)	–0.86	344.5	<.001

	researcher	42.16 (14.22)	–0.86	467.5	<.001	42.86 (18.22)	–0.78	602.0	<.001

	shoemaker	21.53 (13.47)	–1.00	2.0	<.001	15.07 (17.5)	–0.98	30.5	<.001

	surgeon	28.08 (14.92)	–0.99	38.5	<.001	33.67 (19.68)	–0.91	211.5	<.001

	technician	29.7 (15.29)	–0.99	42.0	<.001	35.1 (20.02)	–0.91	230.5	<.001

	voice actor	48.68 (9.16)	–0.89	357.5	.029	47.18 (13.1)	–0.83	478.5	.023

	watchmaker	29.06 (16.3)	–0.98	52.5	<.001	20.6 (21.73)	–0.87	201.5	<.001

Feminine/Neutral	singer	52.48 (8.82)	–0.59	1398.5	.003	47.63 (14.58)	–0.79	604.5	.052

	translator	55.89 (11.12)	–0.26	2496.5	<.001	49.23 (17)	–0.72	811.5	.337

Neutral/Feminine	educator	48.65 (15.49)	–0.43	1879.5	.500	58.91 (20.41)	–0.26	2178.5	<.001

	specialist educator	49.1 (18)	–0.43	1935.0	.652	56.42 (22.48)	–0.38	1812.5	.011

Masculine/Neutral	comedian	47.45 (8.05)	–0.87	428.5	<.001	50.28 (15.78)	–0.71	852.5	.839

	writer	46.1 (10.49)	–0.84	554.5	<.001	48.99 (18.39)	–0.59	1178.0	.568

Neutral/Masculine	rider	47.91 (19.95)	–0.38	1894.5	.660	14.84 (16.03)	–1.00	5.5	<.001

Masculine/Feminine	cook	34.96 (12.37)	–0.96	136.0	<.001	63.54 (24.63)	–0.06	2622.0	<.001

	pastry chef	40.77 (13.54)	–0.84	550.0	<.001	71.84 (22.09)	0.31	3850.0	<.001


On the list of 56 occupations, we then realized non-parametric two-tailed one-sample Wilcoxon signed-rank tests, with a test value of 50 %, for each occupation in each country. Detailed results for the 56 occupations tested in each language are provided in Table 1. Results showed that 13 occupations were consistently associated with feminine gender stereotypes in both the French and Brazilian samples, whereas 34 occupations were consistently associated with masculine gender stereotypes in both samples. However, 9 occupations differed in their perceived gender stereotypes across countries.

### 2.7 Data anonymisation and ethical issues

Data was collected in compliance with the GDPR. The study was completely anonymous. In particular, IP addresses of participants were never collected. To distribute the questionnaire, we used mailing lists to maintain the anonymity of participants. Indeed, at no time did the researchers have access to the participants’ identities or email addresses.

No ethical approval was obtained for the data collection, as it was not required under the applicable institutional and national regulations for this type of fully anonymous study.

### 2.8 Existing use of data

To date, there are no publications or productions based on this dataset.

## (3) Dataset description and access

### 3.1 Repository location

DOI: osf.io/j86z4.

### 3.2 Object/file name

- Primary data: NeFeLaE_final_dataset.xlsx
- Codebook for primary data file: CB_NeFeLaE_final_dataset.pdf
- List of occupations tested: NeFeLaE_occupations_list.pdf
- Mean perceived femininity ratings: NeFeLaE_occupation_means.pdf
- JASP script for t-tests (French sample): NeFeLaE_t-tests_France.jasp
- JASP script for t-tests (Brazilian sample): NeFeLaE_t-tests_Brazil.jasp
- JASP script for correlations: NeFeLaE_correlations.jasp
- Excel file for outlier detection: NeFeLaE_outlier_detection.xlsx
- Codebook for outlier detection: CB_NeFeLaE_outlier_detection.pdf
- Qualtrics script (In French): NeFeLaE_FR_Qualtrics_export.qsf (also available .pdf)
- Qualtrics script (In Portuguese): NeFeLaE_BR_Qualtrics_export.qsf (also available .pdf)

### 3.3 Language

The data is stored in English, French and Portuguese.

### 3.4 License

The open license under which the data has been deposited is Creative Commons Zero (CC0).

### 3.5 Limits to sharing

*N/A*

### 3.6 Publication date

2026/07/07

### 3.7 FAIR data/Codebook

The dataset follows FAIR principles. Regarding Findability, the dataset is publicly available through the OSF repository and associated with a persistent identifier, allowing it to be located and cited. Regarding Accessibility, the files can be freely retrieved from OSF without access restrictions. Regarding Interoperability, the dataset is accompanied by a detailed codebook describing the data structure, variables, coding procedures, and values, facilitating interpretation and use of the data. Regarding Reusability, the documentation provided enables other researchers to understand, verify, and potentially reuse the data and analyses.

The exhaustive codebook for the primary data file “NeFeLaE_final_dataset.xlsx” is available in the document “CB_NeFeLaE_final_dataset.pdf”. It includes for each tab: (1) a general description of the tab (2) a table indicating column letter, name of column, and description. Column descriptions explain the type of data in the box, how they were obtained and computed, and the possible values.

## (4) Reuse potential

The dataset presented here holds significant potential for reuse across several research fields, including psycholinguistics, sociolinguistics, gender studies, cross-cultural research, and language education. It provides normative ratings of the perceived masculinity and femininity of 56 occupational role nouns with distinct masculine and feminine grammatical forms, collected from adult participants in France and Brazil. By focusing on grammatically gendered occupational role nouns in both French and Brazilian Portuguese, the dataset provides a valuable resource for research on grammatical gender, occupational stereotypes, and gender-fair language.

### 4.1 Strengths of the Data

A major strength of this dataset lies in its cross-linguistic design. It provides parallel normative ratings for occupational role nouns in French and Brazilian Portuguese, two grammatical-gender languages, thereby enabling direct comparisons across linguistic and cultural contexts.

Another strength is the careful selection of occupational role nouns with distinct masculine and feminine grammatical forms in both languages. This makes the dataset particularly suitable for research on grammatical-gender languages, occupational stereotypes, and gender-fair language, especially studies investigating the cognitive effects of refeminization. Because the ratings reflect the perceived masculinity and femininity of occupations rather than estimated gender ratios, the dataset also complements previous norming resources and broadens the range of materials available for psycholinguistic research.

### 4.2 Limitations to Consider

Despite its strengths, the dataset has several limitations. First, the sample is restricted to adult speakers from France and Brazil, which may limit the generalizability of the findings to other age groups, regions, or French- and Portuguese-speaking populations outside these countries. Second, the ratings are based on explicit judgments of perceived genderedness and may therefore not fully capture implicit or automatic gender representations.

Another limitation concerns the adaptation of the response format. Although the continuous 0–100 scale used in the present study allows perceived occupational genderedness to be assessed along a masculinity-femininity continuum, this format was inspired by previous norming studies that used continuous ratings to assess perceived gender ratios. Because the present study applied this continuous format to a different construct, some participants may have interpreted the numerical anchors or endpoints differently than intended. Future research could compare this approach with alternative response formats specifically designed to assess perceived occupational masculinity-femininity.

### 4.3 Applications for Reuse in Research and Academia

*Experimental Stimulus Selection*. The dataset provides normative ratings for grammatically gendered occupational role nouns with distinct masculine and feminine forms in French and Brazilian Portuguese. It can therefore be used to select, match, or control experimental stimuli in studies investigating generic masculine, gender-fair language, grammatical gender, or occupational stereotypes.

*Comparison with Existing Occupational Norms*. The dataset complements previous norming studies based on estimated gender ratios (e.g., Gabriel et al., 2008; Misersky et al., 2014) by providing ratings of perceived masculinity and femininity. Combined with previous norming studies, the dataset may notably help identify occupations for which perceived genderedness differs from estimated gender ratios. Such discrepancies could provide valuable insights into systematic asymmetries in occupational gender representations and highlight occupations for which perceived masculinity and femininity extend beyond what would be expected from estimated gender ratios.

*Cross-linguistic and Cross-cultural Studies*. The parallel datasets collected in French and Brazilian Portuguese enable direct cross-linguistic comparisons between two grammatical-gender languages. Beyond the comparison between France and Brazil, the dataset enables future comparisons across national varieties of the same language. For example, similar norming studies conducted with speakers from other French-speaking (e.g., Belgium, Switzerland, or Canada) or Portuguese-speaking countries (e.g., Portugal) could be compared with the present dataset to investigate the extent to which cultural context influences the perceived genderedness of occupational role nouns.

*Gender-fair Language Research*. The dataset is particularly suited for studies investigating gender-fair language. By providing normative ratings for occupational role nouns with distinct masculine and feminine forms, it facilitates the selection of materials for experiments examining the cognitive consequences of refeminization and other gender-fair language strategies.

*Methodological Research*. Gabriel et al. (2008) reported that the orientation of the response scale influenced participants’ judgments of occupational gender. Building on this finding, the present study counterbalanced the direction of the response format. The dataset can therefore be reused to investigate anchoring effects in judgments of occupational genderedness and to inform the design of future norming studies.

### Collaborative and Interdisciplinary Potential

The dataset may facilitate interdisciplinary collaborations between psycholinguists, sociolinguists, cognitive psychologists, and researchers in gender studies interested in the cognitive and social consequences of grammatical gender. Its standardized methodology and openly available materials also make it suitable for replication and extension to additional languages and cultural contexts.

In particular, the explicit rating procedure for perceived masculinity and femininity of occupational role nouns can be readily adapted to other grammatical-gender languages (e.g., Spanish, Italian, or German), thereby facilitating harmonized data collection and future cross-linguistic comparisons.

## (5) Conclusion

This dataset offers a valuable resource for researchers and professionals seeking to understand and address gender representations and stereotypes in language across cultures. Its focus on grammatical gender and profession-related stereotypes, combined with its bilingual, bicultural scope, makes it uniquely positioned to inform both theoretical and applied work. While there are limitations in scope and representation, the structured and replicable nature of the data collection process allows for a wide range of secondary uses, from theoretical exploration to practical applications in education, AI, and public policy.
