# Supplementary material for: Data From the NeFeLaE Project: “Cross-Cultural Evaluations of Grammatically Gendered Occupational Terms: Gender Representations From French and Brazilian Adults”
Source: J Open Psychol Data. 2026 Nov 12;14:9. doi: 10.5334/jopd.146 (PMC13761772; doi:10.5334/jopd.146)
Supplement: Supplementary file 1 [file jopd-14-146-pr1.pdf]

PEER REVIEW FOR "Data from the EPIGMA-EI project: "Cross-Cultural Evaluations of Gendered Occupational Terms: Gendered representations from French and Brazilian Adults"

Dear Morgane Burnel, Catherine Esnard, Rebeca Grangeiro, Lauriane Véron-Delor,

Thank-you for your patience whilst we've secured the feedback of two researchers who have both provided substantive feedback. After review, we have reached a decision regarding your submission to Journal of Open Psychology Data, "Data from the EPIGMA-EI project: "Cross-Cultural Evaluations of Gendered Occupational Terms: Gendered representations from French and Brazilian Adults"". Our decision is to request revisions of the manuscript prior to acceptance for publication.

The full review information is included at the bottom of this email. Please note that there may also be a copy of the manuscript file with reviewer comments available once you have accessed the submission account. We ask you to please consider the following issues and revise the file accordingly. In principle, both reviewers see merit in the work completed but require more detail and precision, especially surrounding the methodology. There are also some questions surrounding theory, framing, and existing works in this space which all warrant further attention. As such, please can I ask that you reflect upon all this feedback you have been provided and work systematically through them to decide what you action and how. As you do so, please provide a clear response to each as per the instructions below.

Kind regards,

Prof Thomas Rhys Evans

Reviewer 1:
Recommendation: Revisions Required

Comments to the author(s)

In this manuscript, the authors describe a dataset on perceived masculinity vs. femininity of different professions in 2024. Data were collected from French speaking adults in France and Portuguese speaking adults in Brazil.

Overall, I found the manuscript very well and clearly written. I can see a lot of value in these data, and thus, the dataset is definitely suitable to be published at JOPD. While reading the manuscript, I have made a few remarks, which I list below. I have also noticed a few typos and grammatical errors, which I corrected directly on the manuscript (see attached separately).

However, before going into details, my major concern is data accessibility. The authors seem to provide the doi (DOI 10.17605/OSF.IO/2Q9E3) but it's not a link. When I searched this doi, I could not find the data. When I searched the title of this manuscript, I could not find the data either. Thus, I could not review the dataset and its accessibility, meaning, this manuscript would need a second round of review.

Now, regarding some questions I had while reading the manuscript:
- On page 2 (introduction), the authors added an interesting quote: Dans la pièce, des femmes[fem.] et un vieux fauteuil[masc.] étaient silencieux[masc.]" (i.e., "In the room, women[fem.] and an old armchair[masc.] were silent[masc.]"). The sentence is rather odd, so I was wondering if it came from any specific source. In that case, I would add a reference.
- On page 3 (introduction), I found the paragraph on how words can be made to appear less strongly gendered very interesting. In addition to the methods mentioned by the authors, I wonder what they think about using different endings, for instance, I have frequently seen étudiant·e·s or étudiantex, the latter also incorporating the non-binary third gender. Is there any literature on the use of such forms? I think it would be a good addition to complete the examples already provided by the authors.
- On page 4, section 2.1 (method section), the authors write they used within-group design. I don't think this is accurate since it seems they had different participants rating French words and other participants rating Portuguese words. Thus, I believe the authors used 'mixed design'. They could then clarify they used a within-group design within each language.
- Page 4, section 2.3 (testing location), I was wondering if the data were collected online, which appears to be so since the authors used Qualtrics. I think this should be clearly mentioned in the section. also, the authors state they collected data from French and Brazilian participants. Were these participants currently living in France and Brazil, or originally came from these countries? If participants were recruited online, how did the authors ensured that participants from other countries did not

participate (e.g. I imagine in France there are native French speakers from Belgium or Switzerland)? How long did the participants live in the respective countries? Also, I do not remember if the authors checked on language fluency. Do we know if these participants were native speakers or fluent non-native speakers? How proficient were they in the tested languages? I understand that the authors might not have answers to all of these questions, but I would appreciate some transparency, for instance, saying we did not collect this and this information.
- Page 5, section 2.6 (quality check) – I was a bit surprised to read about the statistical tests and the fact that 10 occupations were excluded. I understand that the authors may wish to exclude them for their subsequent analysis, but here, in the data paper, I would expect to find all the ratings. Other people who wish to use the data can decide for themselves how to analyse it. Thus, I do not think the exclusion paragraph is necessary. Likewise, table 1 could be simplified, leaving only the tested words (and maybe some of the key information). I would not mark the words in grey that the authors will exclude in future analysis.
- Page 5, section 2.7 (ethical issues) – the authors state: “No ethical approval was obtained for the data collection because it was completely anonymous.” Unfortunately, I don’t think this is how it works. Collecting anonymous data does not allow researchers to conduct unethical experiments (not that I am saying the current experiment is unethical). I believe it depends on the laws of the researchers’ universities whether ethical approvals are required or not. If they are not required, then the authors could state “No ethical approval was obtained for the data collection, as it was not required by the local law”. If ethical approval is required by the local law, then the authors have a problem.
- Pages 6-8 (reuse potential) – I personally find this section the weakest and least well elaborated. To begin with, there are no citations to previous studies, thus, not embedding the suggestions for future in what has already been done. In general, I find this section quite generic, as if it could apply to many different studies, as if it was not written specifically for these data. When reading, two suggestions came to my mind. First, I wonder if these data could be compared to the data from native speakers in other countries (e.g., French data compared to Swiss, Belgian, or Canadian; Portuguese data compared to data from Portugal and other Portuguese speaking countries). As a reader, I would be curious to know if similar datasets already exist in those other countries. I would also be curious to know what other datasets exist on gendered professions, in any languages and countries, to which the current one could be compared to. Overall, this section would benefit from a bit more thought.

That said, I would like to congratulate the authors for a well-written manuscript and believe that all issues can be addressed during revision.

Reviewer 2:
Recommendation: Revisions Required

Comments to the author(s)

The authors present a dataset of gender stereotypical estimations related to 56 role nouns presented to French (121 participants) and Brazilian Portuguese (115 participants) speakers. I believe that the dataset is relevant to the discussion of gender and language, and the paper and dataset are generally well prepared, but there are a number of points that I would like to see addressed.

Major points

1) In relation to the conceptualisation of the stimuli, the authors (using grammatically gendered role nouns as their stimuli) argue that the masculine and feminine forms be distinct from each other in written and phonological form, with this being one of the three ways in which their stimuli differed from Gabriel et al. (2008) and Misersky et al. (2014). The issue here is largely one of framing, so hopefully shouldn't be too difficult to address; Misersky et al. (2014) selected role nouns in their standard forms, which includes a lot of nouns that were not in the 'standard' grammatical gender form (e.g., being instead in the common noun or hybrid/generic noun forms discussed in Gygax et al., 2019, and Tibblin et al., 2022), while it is clear that, while unstated, the current article is only interested in the grammatically gendered form. As such, I would suggest that the authors strengthen their discussion of neutralisation vs. feminisation/grammaticalisation in the previous paragraph to specify that the article follows the feminisation approach, which will then allow the discussion in point three of how the current dataset differs from Gabriel et al. (2008) and Misersky et al. (2014) to be simplified, as the authors can just quickly specify that, in line with the feminisation approach, only grammatically gendered roles with phonologically distinct masculine and feminine forms were selected. I feel that including this specification of grammatical form will help strengthen the relevance of the article for researchers in the gender and language space, as it will allow them to more quickly and accurately evaluate whether, and how, the findings may be relevant for their own work.

2) Further to the last point, the three topics of discussion in this section are scale direction, key question change, and phonetically different masculine/feminine forms. I believe that the authors need to discuss scale format, likely in place of key question change; in Gabriel et al. (2008) and Misersky et al. (2014), gender ratio was used as a stand-in for gender stereotypicality (which has been found to be generally acceptable; e.g., Kim et al., 2024), and with participants tasked with estimating the extent to which the groups are 'actually' made up of women or men (on an 11-point scale that shifted in increments of 10% between 100% male, 0% female and 100% female, 0% male), without being influenced by thoughts about how the groups 'should be'. This phrasing is also free of any potential social desirability bias, so the presentation of the 'new' question as differing in this regard does not make sense to me. In the current paper, the authors have moved away from gender ratios and instead focus on whether roles are perceived as more masculine or feminine, while keeping the same answer scale. I think it would be fair enough for them to specify

that they have kept the spirit of a question that avoids evoking social desirability bias, but implying the previous papers did not do so is incorrect.
3) Further to these points again, I believe that there needs to be a proper justification for why the percentage-based Likert scale was kept when the question no longer matches it. The authors also don’t specify the number of items in the scale; is it a traditional 5- or 7-point scale? Or 11, in keeping with Gabriel et al. (2008) and Misersky et al. (2014)? I would expect to answer the question ‘Is X role perceived as rather masculine or rather feminine’ on a two-point (masculine, feminine), three-point (masculine, neutral, feminine), five-point (very masculine, masculine, neutral, feminine, very feminine), or seven-point (very masculine, masculine, slightly masculine, neutral, slightly feminine, feminine, very feminine) scale, and would be at least a little confused to be presented with an 11-point percentage-based scale. I understand that the percentage based scale is much simpler for analysis, as the ‘steps’ between each of the points is theoretically identical, but I would generally expect the question to align with the scale. This is especially true as the question doesn’t focus on the ratio element; what is the relevance of the 0, 50, and 100 labels for the question as written? For the gender ratios used in the previous papers it made sense, and I can see that the authors use it for calculating %-based ‘mean feminity’ in their results, but due to the mismatch between question and scale, it is likely that participants have been more conservative in their responses, and have answered closer to the midpoint of the scale than they would have otherwise. If there is no clear rationale for this, then I would strongly suggest mentioning this gap in the 'limitations to consider' section, so that readers considering using the data are aware.
4) In section 2.6 it appears that when the authors state at the start of their background section that this is a ‘list of occupations clearly perceived as masculine or feminine, in both French and Brazilian Portuguese’ that they will deselect roles that are masculine in one language and feminine in another; while these aberrant roles may be immaterial to the follow-up study the authors mention, such strong disagreements are very interesting in a dataset like this, so it is odd to remove them. Further, there is no discussion in this section of quality control relating to the participants; even if no one was deselected it would have been good to have, for example, a comment on if any participants were removed due to being non-native speakers etc. Also, since scale direction was controlled for, along with the correlation analysis between languages I would like to see within-language correlations based on scale direction. This removal becomes more confusing as, in the section ‘Applications for reuse in research and academia: cross-linguistic and cross-cultural studies’, the authors suggest using the dataset to examine ‘similar grammatical systems yield[ing] different social outcomes’; I do not follow how this could be done, if the items that differ in terms of stereotypicality are not kept in the dataset.
5) While the ‘pre-deselection’ data is shown in Table 1 in the article, no ‘finalised’ list is shown, and the dataset itself contains raw data, scripts, and the codebook but no easy-to-access results. Further, Table 1 has the role nouns only in Brazilian

Portuguese and French without an English translation. I would suggest, in keeping with previous datasets (e.g., Gabriel et al., 2023), that two .pdfs are added to the OSF dataset; the first being the role noun list (English, French, Portuguese; maybe even two columns for French and Portuguese to show the feminine and masculine forms, as the specific terms used may differ between different populations of French and Portuguese speakers), and the second being the by-item results (Role Noun in column one, then French and Portuguese each having three columns; M, N, and (SD)). This will also heighten the reuse potential, as it makes the dataset more accessible to Bachelor's/Master's thesis students.
6) Section 3.9: The authors briefly state that the data follows the FAIR guidelines, and mention the codebook in their OSF dataset; it is not enough to state that the guidelines are followed, individual discussions of the Findability, Accessibility, Interoperability, and Reusability must be included, even if, for example, the Accessibility statement is that the data is stored on the open access repository at OSF, which allows anyone to retrieve the files following standard protocols.

Minor points
1) The discussion of how the work was adapted from Gabriel et al. (2008) and Misersky et al. (2014) could generally be clearer; point one is in agreement with the previous papers, while (ignoring major issue 2 above) points two and three are (at least stated to be) different from it. I think that the authors may benefit from starting with a brief overview of the existing paradigm, then a statement on what was kept, followed by a statement on what was changed.
2) While I understand what the authors mean with the term 'gendered language', in line with the increasing standardisation of terms in the area, I would suggest using the more specific term 'grammatical gender languages'; this would align with the categories outlined in the Gygax et al. (2019) paper already referenced, and I believe the term's increased accuracy is helpful for those not familiar with the existing discussion around gender and language.
3) Section 2.4: standardise; either male, female, non-binary or m, f, n-b when reporting both Brazilian and French samples.
4) Section 2.5: For clarity, and in line with my comments above, I would suggest updating the discussion of selection criteria to something like "The occupations were selected based on the criteria of both (1) being masculine- or feminine-stereotyped (based on previous research, e.g. …), and (2) being grammatically-gendered roles with unique masculine and feminine forms. The exact…"; the authors have already discussed the form of the roles in their introduction, but where the stereotypicality (or typicality?) ratings of the roles that underpinned their selection came from would need to be specified – were these selected based off the gender ratio findings of Misersky, for example.
5) Section 2.7: should be 'IP addresses were never collected' (rather than was).
6) Section 3.7: Suggest using N/A rather than 'not concerned'.
7) 'Limitations to consider': representations, rather than representatins.

8) ‘Applications for reuse in research and academia: perceived and actual gender roles’: gender stereotypes are a form of gendered representation, and research into gender ratios and gender stereotypes has found that perceived and real gender ratios are closely aligned, and that perceived gender ratios and perceived gender stereotypes are similarly closely aligned. As such, a more tight focus on examining the rare cases where they diverge, rather than on the generalities of if they diverge, might be a much stronger argument for reuse.

9) Throughout the paper the authors use the term ‘feminity’; this is quite a rare term, ‘femininity’ may get the point across more cleanly, especially to a general audience. Regardless, some uniformity is required, as the term ‘femineity’ is also used in the header of Table 1.
